# Evolution of nutritional status and associated factors among formula-fed infants with cow’s milk protein allergy in a government program

**DOI:** 10.1186/s13690-023-01094-3

**Published:** 2023-05-12

**Authors:** Giuliana Rizzo Taveira, Carolina Dadalto Rocha Fernandes, Yasmin Franco Rodrigues Silva, Maria Clara Barcelos de Aquino, Ana Carolina Menezes Vieira da Silva, Carolina Perim de Faria, Míriam Carmo Rodrigues Barbosa

**Affiliations:** 1 Federal University of Espírito Santo, Vitória, Brazil; 2 University of North Florida - Jacksonville, Flórida, USA

**Keywords:** Public policies, Public health, Cow’s milk protein allergy, Milk substitutes, Infant formula, Nutritional status

## Abstract

**Background:**

Cow’s milk protein allergy (CMPA) is a common allergy in infants and can affect proper growth and development. This study verified factors associated with the evolution of the nutritional status (NS) among infants with CMPA fed with hypoallergenic formulas (HF).

**Methods:**

This is a longitudinal study of infants (*n* = 1036) participating on a governmental program in Brazil. Researchers assessed Nutritional status before HF treatment (T1) and after HF treatment (T2). The causality of exposure variables on the evolution of NS was verified by Multinomial Logistic Regression (MLR).

**Results:**

We observed an increase in anthropometric indexes analyzed with statistically significant results (*p* < 0.01). The weight/age and height/age scores showed a significant reduction in infants with nutritional deficit. The Body Mass Index (BMI) showed a decrease in the number of infants with nutritional deficit (< -2 z-score). On the other hand, there was an increase in those classified as at risk of overweight, overweight and obese. MLR showed that those who remained < 12 months in the program had a lower odds ratio (95% CI = 0.355–0.906; *p* = 0.018) to have inadequate NS with increasing BMI. Preterm infants were 4 times more likely (CI 95% = 1.520–10.694; *p* = 0.005) to have their BMI decreased and those who received nutritional counseling had a lower odds ratio (CI 95% = 0.411–0.953; *p* = 0.029) to maintain adequate NS.

**Conclusion:**

The program has a significant impact on the NS of infants with CMPA. The constant management and implementation of differentiated criteria according to the evolution of NS for the supply of HF is fundamental in the continuity of this public policy.

## Background

Food allergy is currently considered a public health problem due to an increase in diagnosis over the last few years. Cow’s Milk Protein Allergy (CMPA) is one of the most commonly observed allergies in the first year of life [1–5]. The gold standard method for diagnosis is the elimination and reintroduction of the allergen called Oral Food Challenge (OFC) [2]. Treatment includes a strict diet for nursing mothers and hypoallergenic formulas (HF) for formula-fed infants. Infants with food allergy tend to have growth problems and nutritional deficiencies [6–11]. The nutritional consequences may be even more evident in infants with CMPA since this age group tends to have dairy diets. Furthermore, other factors can influence the growth problems of these infants, such as late diagnosis, early development of the disease, multiple allergies, as well as excessive and unnecessary food restrictions [12]. Public policies are essential to ensure adequate development of infants in risk of developing growth problems, especially in underdeveloped countries [12].Regarding the excessive cost of HFs to manage CMPA, in BRAZIL, government programs have been created to assist this population [13]. Few studies, however, have been published on the nutritional and clinical status of CMPA infants fed with HFs provided by these programs [13–15]. Therefore, this study aims to describe the epidemiological profile and analyze the evolution of nutritional status and factors associated of formula-fed infants through critical reflection and, if necessary, proposition of new strategies for the conduction of public policy.

## Methods

### Study design/population

A retrospective longitudinal study with secondary data which evaluated the clinical profile and the anthropometric evolution of HF-fed infants with CMPA who take part in a program designed by the Brazilian Unified Health System (called “Sistema Único de Saúde”- SUS) and funded by the government Espirito Santo Department of Health (DOH-ES), Brazil focused in providing HF. Inclusion criteria for the program are the presentation of a standardized form called Request for Nutritional Formula (“*Laudo para Solicitação de Fórmulas Nutricionais”* – LFN), which contains clinical and anthropometric data, which must be renewed by prescribers (pediatricians) every 3 months to continue treatment.

The program receives children from 0 to 24 months of age who can be part of it until they reach 24 months. For the analyses, the children were categorized according to their time in the program, those who remained in the program for less than 12 months and those who remained in the program for a period of 12 to 24 months. This study included infants who accessed the program in 2017 and 2018, and were able to receive formula until 2020. Infants with malabsorption syndromes, neurological disorders, tube feeding, genetic syndrome, inborn errors of metabolism, neoplasia, preterm with corrected gestational age of less than 40 weeks, and infants who requested HF only one time or with forms missing anthropometric data were excluded.

To classify the nutritional status of preterm infants, the gestational age was corrected to 40 weeks. All infants received the formula for a minimum of 3 months to a maximum of 27 months.

The program’s eligibility criteria was CMPA diagnosis reported by pediatricians, which means that the infants had symptoms after a strict allergen diet followed by reintroduction of the allergen or clinical’s diagnosis. The assistant pediatricians, were responsible for all formula prescriptions and infant’s follow up.

The HF provided in this study are extensively hydrolyzed formula (EHF) and amino acid-based infant formula (AAF), according to medical report or severity of clinical manifestation.

### Data collection

Data were collected from the documental analysis of medical and/or dietitian reports included in the electronic medical files of DOH-ES patients, regarding two moments. The first moment (T1) refers to the form that initiated the request process and the second moment (T2) refers to the form of the last documentation requested, which characterizes the beginning and the end of treatment with the HF provided by the State, respectively.

The instrument used by DOH-ES is the LFN form, from which anthropometric information, feeding information, and clinical history were obtained. In addition to the LFN form, the growth charts attached to the requests confirmed the anthropometric data.

### Exposure variable

Epidemiological and clinical data: infant’s age at program entry (categorized in 2 groups from ≤ 12 months old and > 12 months old); gender; classification of gestational age at birth; twin birth; duration of exclusive breastfeeding (EBF); dietary patterns at the beginning of symptoms; origin of prescription (via SUS—public or private), nutritional counseling; classification of symptoms according to the system involved (gastrointestinal, skin, and respiratory); and formula requested: EHF or AAF.

Anthropometric variables: Weight (kg); Length/Height(cm); nutritional status was considered according to the WHO growth indicators [16]; and evaluation of the z-score values through the reference ranges for the weight/age (W/A), height/age (H/A), weight height (W/H), and BMI/age (BMI/A).

### Outcome variable

The evolution of nutritional status was conducted by analyzing the differences between the categories of anthropometric index of BMI/age at T1 and T2; infants were then reclassified into 4 new categories, according to the type of evolution they presented during treatment, using normal weight as the standard reference [17].

The 4 new categories were: 1) Catch up Growth (CUG)– infants who had growth indicators (GI) below the normal weight range (< -2 z-score and >  + 1 z-score) at T1, and at T2 had within the normal weight range (≤ -2 z-score and <  + 1 z-score). 2) Excessive Weight Gain (EWG) – infants whose growth indicators (GI) were within the normal weight z-score at T1, and at T2 with an increase in the z-score with values higher than those of normal weight (> + 1 z-score). 3) Growth Inappropriate (GINP) – infants who had NS classified within the normal weight z-score at T1, and at T2 had NS with a decrease in the z-score with values below those of normal weight (< -2 z-score), or those that at T1 and T2 had NS outside the normal weight values. 4) Growth Appropriate (GAP)– infants who had GI classified within the normal weight z-score at T1 and T2, therefore maintaining adequate NS.

### Statistical analysis

Statistical analysis was performed using Statistical Package for the Social Science (SPSS), version 20.0. Differences were classified as statistically significant for all association tests at a level of 5% (*p*˂0.05). Numerical variables were evaluated for normality by the Kolmogorov–Smirnov test, and bootstrapping procedures were performed (1000 resampling; 95% CI BCa) [17].

The comparison of the proportions of anthropometric indexes (W/A; H/A; W/H; and BMI/A) at T1 and T2 were performed using the Wilcoxon test. To explain the effect and verify the causality of the exposure variables on the outcome variables, Multinomial Logistic Regression (MLR) was performed, using the CUG category as a reference group resulting in odds ratios with a 95% confidence interval.

The DOH-ES approved the study and granted permission to use the data, as did the Ethics and Research Committee on Human Beings (CEP) of the Federal University of Espírito Santo under CAEE:: 39716120.2.0000.5060 and technical advice nº 4.432.029.

## Results

This study included 1036 infants as participants. (Table 1) Our results show higher percentage of males than females, and most of the physicians’ prescriptions were from private services. The median duration of exclusive breastfeeding was 24.4 days (IQR 0 to 84.3; *p* = 0.0446) and this data was observed in 68.4% of children (*n* = 709). Furthermore, most infants were diagnosed with CMPA after supplementing breastmilk feeding with formula. Insufficient breast milk (38.7%) was the main reason for the introduction of infant formula.

**Table 1:** Characteristics of infants with cow’s milk protein allergy who were fed hypoallergenic formula provided by the government program

**Sex** **n (%)**	
Female	460 (44.4)
Male	576 (55.6)
**Twinsbirth n(%)**	
Yes	38 (3.7)
No	998 (96.3)
**Preterm birth n (%)**	
Yes	61 (5.9)
No	975 (94.1)
**Dietary patterns at the begin of symptoms n**^**a **^**(%)**	
Exclusive breastfeeding	149 (28.4)
Infant formula-fed	375 (71.6)
**Type of medical prescription n (%)**	
Private	822 (79.3)
Public	214 (20.7)
**Number of systems affected n (%)**	
1 system	638 (61.6)
2 systems	329 (31.8)
3 systems	69 (6.7)
**Duration in the program n (%)**	
≤ 12 months	575 (55.5)
> 12 months	461 (44.5)
**Age of the infant at T1 n (%)**	
0—6 months	785 (75.8)
6—12 months	190 (18.3)
> 12 months	61 (5.9)
**Infant formula requested n (%)**	
EHF	646 (62.4)
AAF	390 (37.6)
**Nutritional Counseling**	
Yes	317 (30.6)
No	719 (69.4)
Age at end of program treatment. months (SD)	14.32 (± 6.14)
Exclusive breastfeeding period. months (SD)	1.5 (± 1.825)

^a^*N* = 524;*SD* standard deviation, *T1* moment one, *T2* moment two, *EHF* extensively hydrolyzed formula, *AAF* amino acid-based formula

Most children started using hypoallergenic formulas before 6 months of age (75.8%). The median ages in T1 was 3.65 months old (IQR 2.08 to 5.80) and in T2 was 13.63 months old (IQR 9.4 to 19.22) and the median total program duration was 11.19 months (IQR 7.6 to 15.9).

**Fig. 1:**
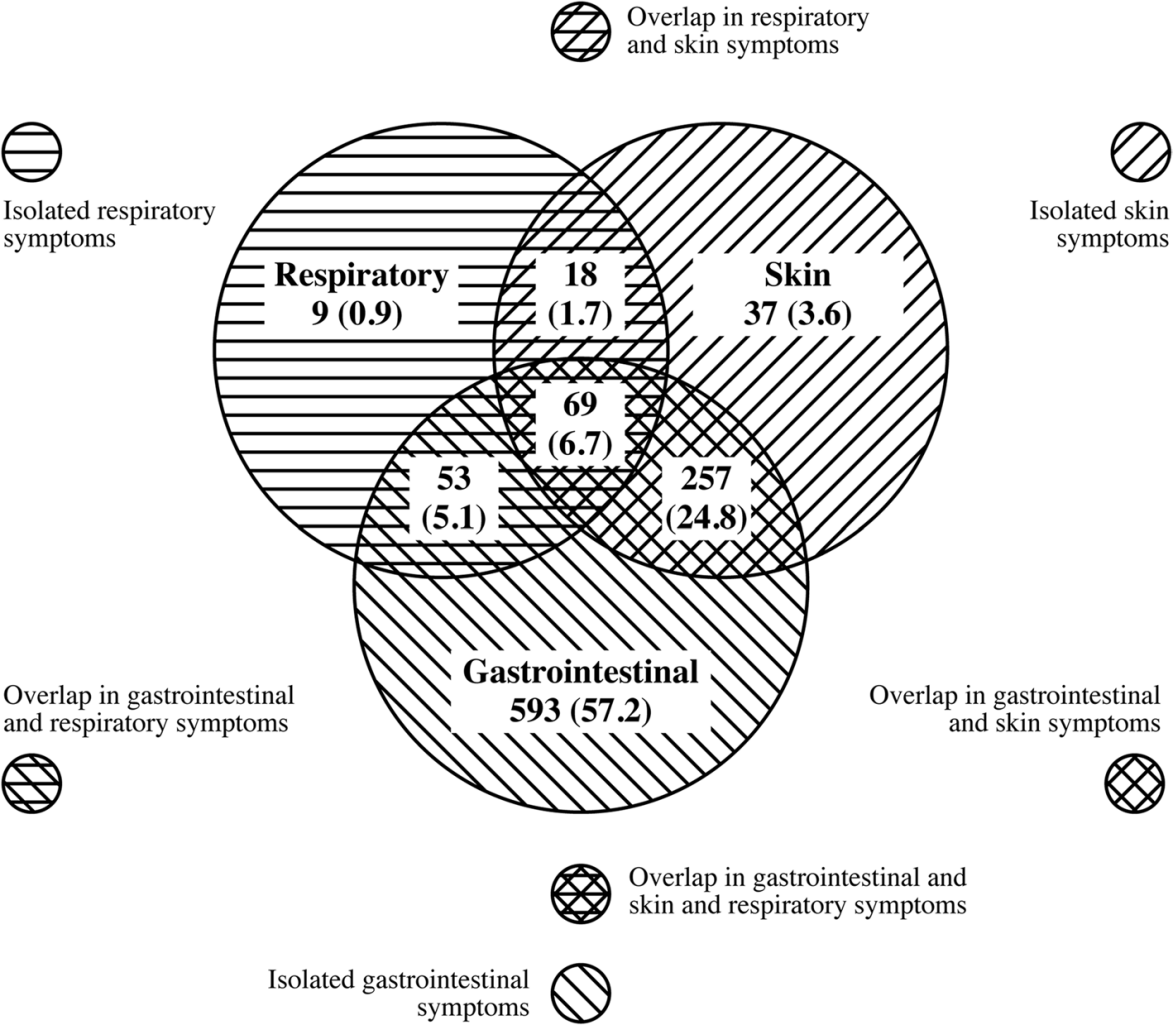
Distribution of the number of cases according to clinical manifestations and the system involved

The (Fig. 1) summarizes the signs and symptoms according to the affected system. 93.8% (*n* = 972) of the infants had gastrointestinal manifestations, followed by 36.8% (*n* = 381) skin and 14.4% (*n* = 149) respiratory manifestations. When evaluated as isolated symptoms, 639 (61.7%) infants had manifestations in only 1 organ system, and the gastrointestinal system manifestations was prevalent in 57.2% (*n* = 593) of the cases. When evaluated together, 31.7% (*n* = 328) of the infants had two systems affected, and the association of gastrointestinal and skin manifestations occurred in 24.8% (*n* = 257). EHF was the formula with the highest percentage of use (62.4%), and only 30.6% of infants received nutritional counseling.

The (Table 2) shows the assessment of NS at both moments (T1 and T2). The comparison of NS between T1 and T2 showed statistical significance through the wilcoxon test for all anthropometric indexes (*p* < 0.001), as expected.

**Table 2:** Nutritional status of infants with cow’s milk protein allergy who were fed hypoallergenic formula provided by the government program

	**T1**	**T2**	p value^*^
**Weight Kg**^a^	6.1 (2.06)	9.91 (2.08)	
**Height cm**^a^	61.3 (0.079)	76.4 (0.079)	
**W/A z-scores**^a^	-0.72 (1.29)	-0.045 (1.15)	*p* < 0.001
**H/A z-scores**^a^	-0.74 (1.56)	-0.198 (1.5)	*p* < 0.001
**W/H z-scores**^a^	-0.16(1.47)	0.25 (1.19)	*p* < 0.001
**BMI z-scores**^a^	-0.43 (1.36)	0.24 (1.24)	
**W/A classification n (%)**			
severely underweight for age	55 (5.3)	8 (0.8)	*p* < 0.001
underweight for age	104 (10)	26 (2.5)	
Appropriate weight for age	864 (83.4)	957 (92.4)	
overweight for age	13 (1.3)	45 (4.3)	
**H/A classification n (%)**			*p* < 0.001
Very low height	73 (7.1)	30 (2.9)	
Low height	102 (9.8)	59 (5.7)	
Adequate height	861 (83.1)	947 (91.4)	
**W/H classification n (%)**			*p* < 0.001
Severely underweight	26 (2.5)	5 (0.5)	
Underweight	47 (4.6)	17 (1.7)	
Normal weight (Eutrophy)	776 (75.5)	779 (75.2)	
Possible risk of being overweight	119 (11.6)	170 (16.4)	
Overweight	41 (4)	49 (4.7)	
Obese	18 (1.8)	16 (1.5)	
**BMI classification n (%)**			*p* < 0.001
Severely underweight	33 (3.2)	9 (0.9)	
Underweight	74 (7.1)	18 (1.7)	
Normal weight (Eutrophy)	811 (78.3)	767 (74)	
Possible risk of being overweight	77 (7.4)	173 (16.7)	
Overweight	28 (2.7)	51 (4.9)	
Obesity	13 (91.3)	18 (1.8)	

^a^Mean *SD* standard deviation, *H/A* height-for-age, *W/A* weight-for-age, *W/H* weight-for-height, *BMI* body mass index

^*^Wilcoxon test (*p* < 0.005)

When comparing the BMI/A in T1 and T2, there was an important reduction in the levels of underweight/severely underweight (72.7%) and maintenance of the normal weight percentage, with a slight reduction (5.43%). On the other hand, there was a significant increase in the number of infants classified with z-score values higher than those of normal weight (Risk of Overweight, Overweight, and Obesity), from 11.38% to 23.35%.

Regarding the evolution of NS, most infants were classified as GAP (*n* = 635), followed by 176 infants in the EWG category, 132 cases in the GCU category, and 93 cases in the GINP category.

After chi-square analysis, there was a significant association (*p* < 0.005) between the evolution of NS and some exposure variables, such as gestational age, origin of medical prescription, presence of cutaneous and systemic symptoms, total time engaged in the program, age at T1, and nutritional counselling.

The variables that presented *p* < 0.20, such as twin birth (*p* = 0.121), gestational age (*p* = 0.005), age at T1 (*p* = 0.001), total time engaged in the program (*p* = 0.002), origin of medical prescription (*p* = 0.030), nutritional counseling (*p* = 0.004), presence of gastrointestinal symptoms (*p* = 0.068), presence of skin symptoms (*p* = 0.023), presence of respiratory symptoms (*p* = 0.176), isolated gastrointestinal symptoms (*p* = 0.018), isolated skin symptoms (*p* = 0.144), association of skin – respiratory – gastrointestinal symptoms (*p* = 0.074), and number of systems according to the symptom (*p* = 0.088), were inserted to the multinomial logistic regression. However, the variables presence of gastrointestinal, skin and respiratory symptoms, isolated gastrointestinal and skin symptoms and association of gastrointestinal-cutaneous-respiratory symptoms and number of systems involved showed mucolinearity and were excluded from the model.

Thus, the final MLR model included the independent variables “twin birth,” “classification of gestational age,” “origin of medical prescription,” “number of systems involved,” “total time in the program,” “age at T1,” and “nutritional counseling,” with the evolution of nutritional status (ENS).In the proposed model, for the variable "classification of gestational age", the classification “pre-term” was compared to “term”. For “number of systems involved”, the classifications of 1 and 2 systems involved were compared to those that had 3 systems involved. For the “total time in the program” children who remained ≤ 12 months in the program were compared to those who remained > 12 months (with a limit of 2 years of age) and finally, for “nutritional counseling” children who had nutritional counseling were compared to those who did not.

The final regression model of the multinomial logistic regression (Tables 1 and 3) for ENS was significant (*p* < 0.001). Statistical significance was observed for the variables of total time in the program < 12 months in the categories for EWG (OR = 0.567; 95% CI = 0.355 – 0.906; *p* = 0.018) and GAP (OR = 0.638; 95% CI = 0.429 – 0.949; p = 0.026). In the GINP category, the significant variables were prematurity (OR = 4.031; 95% CI = 1.520 – 10.694; *p* = 0.005) and presence of only 1 system involved (OR = 0.245; 95% CI = 0.072 – 0.829; p = 0.024); and the presence of nutritional counseling showed significance in the GAP category (OR = 0.626; 95% CI = 0.411 – 0.953; *p* = 0.029).

**Table 3:** Odds ratio between independent variables and evolution of infant with cow’s milk protein allergy who were fed hypoallergenic formula provided by the government program

	EWG		GINP		GAP	
**Variable**	OR (95% CI)	*p* value	OR (95% CI)	*p* value	OR (95% CI)	*p* value
**Infants’ duration in the program**						
>12 months	1					
≤12 months	0.567 (0.355 – 0.906)	**0.018**	1.255 (0.704 – 2.24)	0.441	0.638 (0.429 – 0.949)	**0.026**
**Preterm birth**						
No	1					
Yes	0.696 (0.239 – 2.021)	0.505	4.031 (1.520-10.694)	**0.005**	1.064 (0.465 – 2.432)	0.883
**Number of systems affected**						
3 systems	1					
2 systems	0.486 (0.145 – 1.623)	0.241	0.299 (0.085 – 1.059)	0.061	0.629 (0.211 – 1.879)	0.145
1 system	0.397 (0.124 – 1.277)	0.121	0.245 (0.072– 0.829)	**0.024**	0.454 (0.157 – 1.313)	0.407
**Nutritional Counseling**						
No	1					
Yes	1.05 8 (0.643 – 1.740)	0.825	0.825 (0.452 – 1.505)	0.531	0.626 (0.411 – 0.953)	**0.029**

*CI* confidence interval, *OR* odds ratio. *p*<0.005, *EWG* Excessive Weight Gain, *GINP* Growth Inappropriate, *GAP* Growth Appropriate. The Catch up Growth (CUG) group was used as the reference group

## Discussion

Our study showed the impact of the government program on the evolution of the nutritional status of children with CMPA. We noticed that not only clinical factors can interfere in the final NS, but also access to nutritional professionals and time in the program.

In the evaluation of socioeconomic factors related to the access of beneficiaries of the program, it is noted that more than 70% of the requests came from private prescribers. This result brings a reflection whether this finding could be related to socioeconomic status or to real access to public health services. Studies evaluating access to health technologies provided by the SUS, show similar percentages of private prescriptions [18] having attributed sociodemographic/ socioeconomic status [19] and the smaller supply of public professionals than in the private network, as possible predictors of this difference [18].The insufficiency of doctors or specialists in the public network has been shown to be an important cause of difficulties in referring patients to the programs, as well as maintenance of treatment, especially for those who lived in small towns. This deficiency can impact in the correct diagnosis, in the access to the supply of nutritional formulas provided by SUS [20], affecting directly the proper development of children.

In the anthropometric evaluation the results of weight and height showed nutritional deficits (< -2SD) at T1 (initial) were15.3% for the W/A index and 6.4% for the W/H index, similar results were observed within the literature [10, 21, 22]. We observed the median z-score for W/A in infants at T1 was -0.62 (IQR-1.48 to 0.12), a similar result was observed in a cohort study with 119 infants who were fed hypoallergenic infant formula, in which the mean weight-for-age z-score at baseline was -1.17 [23]. We observed in our study that at T2 there was a significant increase in the z-scores of all anthropometric indexes, as observed in other studies [24, 25], which found a positive difference after the use of EHF in z-scores for W/A and BMI/Age, respectively.

In addition to the increase of the anthropometric indexes, it was possible to identify changes in the NS categories between T1 and T2. The literature has pointed that W/A is the appropriate index to monitor weight gain, being the main one for assessing underweight [26]. The H/A index had an important positive evolution, reducing the percentage of infants with inadequate height from 16.9% at T1 to 8.6% at T2, which is an important indicator for public health policies since it relates to the effect of adverse situations on the linear growth of a child, considered the most sensitive for measuring the quality of life within a population [26]. In 2015, Dupont et al. [23], also observed a significant improvement in the proportions of babies with z-score > -2 SD for weight-for-age and height-for-age, in infants with CMPA who used EHF.

Although the W/A and H/A indexes are essential for the individual follow-up of infants, the analysis of child development must consider the association of both since weight gain and height growth are better evaluated together. Thus, the index analysis that takes this proportion into account, such as the W/H index and the body mass index (BMI), are essential.

The body mass index (BMI) is recommended in Epidemiologic studies diagnosis of nutritional disorders, especially to assess the risk of childhood obesity, in addition it can be used in all stages of life [26, 27]. The results showed a significant reduction in the number of infants classified as severely underweight and underweight at the end of the study (T2), according to the BMI/A index. Approximately 90% of the infants who entered in the program with nutritional deficit, had their z-score increased to levels greater than -2 SD.

The literature is unquestionable about the need to prevent early malnutrition in order to avoid infant mortality [28–30]. Malnutrition can cause significant losses, individually and collectively, by leading to deleterious effects on brain and cognitive development with lifelong consequences [31].

These results suggest that the public health policy of HF supply has a positive impact on the reduction of malnutrition in infants, which strengthens its need and justifies more investment and maintenance.

The same results, however, also showed an increase in infants with risk of overweight and with overweight at T2. Despite few studies showing this trend in infants with CMPA, studies with healthy infants have suggested that faster weight gain is associated with future overweight in those using infant formulas when compared with those who are breastfed [32, 33].

The multivariate analysis showed effect of treatment time < 12 months was significant for the EWG group, suggesting that patients who use nutritional formulas for a period shorter than 12 months are less likely to increase their BMI by higher values than the normal weight group. Considering that our study showed an increase in BMI/A, the protection factor given by the treatment time of less than 12 months may be related to the protection of the increase in z-score for levels beyond normal weight. Our data leads us to question whether these results are due to an inadequate diagnosis of CMPA, which would indicate to the unnecessary use of infant formulas; or if, even with the diagnosis of CMPA, the volume of formula according to age group and NS could be overestimated.

The classification of overweight and obesity in infants with CMPA described in the literature is not common. The use of Public Health Policy without effective control mechanisms may be negatively interfering in the NS of some of the benefited infants. Intervening assertively in these infants, especially in this age group, can prevent overweight disorders in schoolchildren and in adulthood.

For the GINP group, infants “born preterm” and the “number of systems involved” were the variables that significantly affected the model. Prematurity increased by 4 times the chance of infants having inadequate NS or maintaining inadequate NS. These results are consistent with the literature, which has shown that the growth patterns of preterm infants are different from those of full-term infants [32, 34, 35]. It was evident that up to 2 years of age the NS of these infants with CMPA were influenced by gestational age at birth.

Possibly, such manifestations are related to microbiome changes, which have already been shown to be different in premature infants. According to some hypotheses, prolonged hospitalization in a neonatal intensive care unit (NICU) can result to non-physiological gastrointestinal maturation [34].

Infants who remained in the program for less than 12 months were less likely to remain in the GAP group than those who stayed longer in the program when compared to the reference group. This result indicates that time is a decisive factor in the improvement of NS in infants, especially those with nutritional deficiencies.

Infants in the GAP group who received nutritional counseling were less likely to remain in the GAP than those in the reference group, who had their NS adjusted. These results may suggest that the infants who needed it most, that is, those with severely inadequate weight, were prioritized in referral and nutritional care, and infants who started treatment with adequate EN did not receive nutritional counseling with the same frequency.

This hypothesis is supported by the lack of coverage of dietitians/nutritionists in the public health network [36, 37]. Cervato-Mancuso et al., 2012, identified that just over half of the Family Health Support Centers had nutritionists, albeit with unequal distribution throughout the city of São Paulo, prioritizing places with greater social vulnerability and greater demand for nutritional care [38].

Therefore, we observed that of the 4 variables that influence nutritional evolution, “Preterm birth” and the “Number of systems affected” are related to clinical characteristics of CMPA.

The variable “nutritional counseling influences the organizational flow of the program and can even be modified by the government. On the other hand, the variable duration in the program may be related to the organization flow of the program but also to the age when the child was included in the program since they can only remain until 24 months of age.

We noticed that the treatment time influenced the EWG and GAP groups, for the EWG group, remaining in the program for a shorter time (< 12 months) reduced the chances of the child developing excessive weight gain at the end of the treatment In 43% while for the GAP group, it was demonstrated that staying in the program for < 12 months reduced the chances of maintaining adequate NS in 56%.

In addition, we observed that nutritional counseling seems to have been prioritized in the group that needed nutritional recovery, however, it is clear that this counseling needs to be expanded to all groups, including those who started with adequate NS.

Historically, deficit and excess weight have always been treated in completely different ways. These two extremes of nutritional problems, however, are related to the same spectrum of inadequate nutritional contribution. Our results showed a fine line between the improvement in the NS of infants with nutritional deficits and the increase in those with adequate NS after the use of HF. It may be necessary to use different criteria when considering the NS and its evolution through the use of formulas provided by the program.

The limitation of our study w the lack of quantitative analysis of total calories intake, such as joint analysis with other foods consumed by the child, despite all of them were using formulas prescribed by a health care provider pediatrician. Therefore, further studies are needed to assess the adequacy of the prescription and food intake of infants with CMPA.

## Conclusion

The longitudinal analysis of nutritional status presented in this study leads to the conclusion that the access to hypoallergenic formulas significantly affects the nutritional evolution of children. When comparing the Body Mass Index for age (BMI/A) in the first and last moments, there was an important reduction in the levels of thinness and accentuated thinness and maintenance of the percentage of eutrophy. On the other hand, there was a significant increase in the number of infants classified with higher z -score (Possible risk of Overweight, Overweight and Obesity). Factors such as time in the program, nutritional counseling gestational age and the number of organic systems affected by the allergy also influence the nutritional evolution of children with CMPA.

It may be necessary to apply different parameters for the start and continuation of the formula supply, in which the current nutritional status and its evolution must be considered for the program's formula supply to be maintained. Nutritional status follow up should be institutionalized in order to prevent excessive weight gain due to use of special formula. For this, the authors recommend to expand the availability of pediatric professionals and dietitian/nutritionists in the public health network, even considering introducing these professionals into the program, such as in the creation of an exclusive reference center for children with CMPA. We would like to highlight that a close follow up of governmental health programs such as the one discussed in our paper is essential to guarantee not only appropriate care as well as rational use of public funds.

## Data Availability

The datasets used and/or analyzed during the current study are available from the corresponding author on request.

## Abbreviations

CMPA: Cow’s Milk Protein Allergy
BMI: Body mass index
BMI/A: BMI for Age
Cm: Centimeters
CUG: Catch up Growth
DOH-ES: Department of Health of the State of Espírito Santo
EBF: Exclusive Breastfeeding
ENS: Evolution of nutritional status
ES: Espírito Santo
EWG: Excessive Weight Gain
AAF: Amino Acid-Based Infant Formula
EHF: Extensively Hydrolyzed Protein Formula
GAP: Growth Appropriate
GINP: Growth Inappropriate
H/A: Height for age
HF: Hypoallergenic formulas
Kg: Kilograms
LFN: Laudo para Solicitação de Fórmulas Nutricionais
MLR: Multinomial Logistic Regression
NS: Nutritional status
OFC: Oral Food Challenge
SD: Standard deviation
SESA: Secretary of State for Health
SP: Overweight
SUS: Health Unic System (Sistema Único de Saúde)
T1: First Moment
T2: Second Moment
W/A: Weight for age
W/H: Weight for height
WHO: World Health Organization
